# Integrated Navigation Algorithm Based on Multiple Fading Factors Kalman Filter

**DOI:** 10.3390/s22145081

**Published:** 2022-07-06

**Authors:** Bo Sun, Zhenwei Zhang, Shicai Liu, Xiaobing Yan, Chengxu Yang

**Affiliations:** 1 College of Intelligent Equipment, Shandong University of Science and Technology, Tai’an 271019, China; bo_sun@sdust.edu.cn (B.S.); sdustliu@163.com (S.L.); ycxjnj@163.com (C.Y.); 2 College of Electronic and Information Engineering, Shandong University of Science and Technology, Qingdao 266590, China; 3 College of Communication Engineering, Taishan College of Science and Technology, Tai’an 271000, China; yanxb_1975@163.com

**Keywords:** integrated navigation system, Kalman filter, fading factor, state switching

## Abstract

An integrated navigation algorithm based on a multiple fading factors Kalman filter (MFKF) is proposed to solve the problems that the Kalman filtering (KF) algorithm easily brings about diffusion when the model becomes a mismatched or noisy, and the MFKF accuracy is reduced when the fading factor is overused. Based on the innovation covariance theory, the algorithm designs an improved basis for judging filtering anomalies and makes the timing of the introduction of the fading factor more reasonable by switching the filtering state. Different from the traditional basis of filter abnormality judgment, the improved judgment basis adopts a recursive way to continuously update the estimated value of the innovation covariance to improve the estimation accuracy of the innovation covariance, and an empirical reserve factor for the judgment basis is introduced to adapt to practical engineering applications. By establishing an inertial navigation system (INS)/global navigation satellite system (GNSS) integrated navigation model, the results show that the average positioning accuracy of the proposed algorithm is improved by 26.52% and 7.48%, respectively, compared with the KF and MFKF, and shows better robustness and self-adaptability.

## 1. Introduction

KF is a state estimation method with superior performance due to its small data storage and high estimation accuracy, which is commonly used in integrated navigation systems [1]. The KF algorithm is usually applied to the INS/GNSS integrated navigation by developing a mathematical model of the integrated navigation error, the whitening of colored noise is introduced into the KF, which solves the effect of colored noise to improve the accuracy of the navigation results [2]. In the literature [3], a loosely coupled GNSS/SINS/GC/VL integrated navigation system based on KF was proposed to establish the filtering equations using the introduced heading and speed information, which can realize the error correction of SINS after filtering. KF is not only widely used in INS/GNSS integrated navigation, but also has many uses in other positioning models [4,5]. However, the utilization of standard KF requires an accurate dynamic model of the system and noticeable noise characteristics, the filter will not be optimal if there are errors in the model or if there is a severe noise mismatch and the final filtering produces divergence [6,7].

To prevent filtering divergence, a large number of scholars have studied this issue [8,9,10]. Deep learning prediction networks can effectively improve the prediction accuracy by scaling and translating the input learnable parameters [11,12]. However, it is then difficult to apply to practical applications in integrated navigation at present. Variational Bayesian is a commonly used method in industrial control and state estimation [13]. In [14], a variational Bayesian adaptive Kalman filter was proposed to approximate the statistics of the process noise variance of a time-varying system by the joint distribution of the recursive state and noise parameters of the system, but there is the problem of frequent process noise covariance transformations leading to the reduction of filtering accuracy. An improved Sage–Husa adaptive filtering was proposed to suppress the filtering divergence caused by observation noise by continuously updating the observation noise variance, but the algorithm sacrifices a certain filtering accuracy to ensure a stable filtering effect and is not suitable for dealing with the case of inaccurate model establishment [15]. Su et al. put forward a method to use the fading factor matrix and fault detection in one way [16], which is innovative, but the algorithm derivation process is tedious and the matrix elements are not always accurately obtained. An adaptive Kalman filtering algorithm based on innovation was proposed in [17], which continuously updates the observation noise variance, suppresses white noise by collecting estimated and measured variables to process the system noise variance, and suppresses the noise variance caused by environmental perturbations by introducing a fading factor to increase the weight of the current observation variables.

Fading Kalman filtering algorithm is a kind of adaptive Kalman filtering algorithm [18,19,20]. An adaptive filtering algorithm based on the innovation covariance was proposed [21], which construct a function to find the fading factor by constructing the covariance estimate of the innovation sequence with the theoretical value, which is simple to compute and improve the reliability of the filtering algorithm to some extent. However, the single factor fading Kalman filter reduces the estimation accuracy of the filter because it has the same adjustment ability for each channel of the error covariance matrix. The idea of fading filtering is usually to increase the weight of the current observation by increasing the error variance matrix of one-step predicting [22]. However, the computation process of the scalar fading factor is tedious and has the same adjustment capability for each filter channel, which makes the stability and accuracy of the improved filter limited. To overcome the limitation of the low performance of a single fading factor filtering, a method was proposed to adjust each channel with different weights by introducing multiple fading factors [23], which can effectively suppress the filtering divergence when there are sudden changes in system noise disturbances, but multiple fading factors will increase the computational load as the number of calculations increases. Pan et al. constructed a scalar fading factor using an improved observation noise covariance matrix and innovation covariance matrix estimated by exponential weighting, and then calculated multiple fading factors to calibrate the error variance matrix of one-step predicting [24], which is better in terms of filtering convergence speed and filtering accuracy compared to conventional filters.

The selection of the fading factor is the key to fading filtering. The open-window method is a traditional method for obtaining the fading factor, which obtains the fading factor matrix by computing the estimates of the innovation covariance, and the fading factor matrix assigns different weight coefficients to different filter channels. The filtering effect is better than the single fading factor when the system noise increases [25]; however, the estimates of the innovation covariance obtained by the open-window method are less reasonable and prone to misclassification. An innovative method was proposed to improve the role of the newly observation data in the filter by weighting the innovation covariance sequence with exponential decay to provide accurate fading factor for the filter [26], which can obtain great results when the system is modeled inaccurately or when the noise is time-varying. In [27], an adaptive threshold γ for the H-infinite Kalman filter based on multiple fading factors was introduced, which not only enables the filter to converge quickly in the initial stage, but also to adapt to extreme environmental changes.

Although current studies have improved and perfected the solution method for multiple fading factors, the overuse of fading factors in conventional MFKF algorithms in integrated navigation systems not only increases the computational load, but also tends to degrade the filtering accuracy [28]. To overcome this difficulty, adaptive control is widely used [29,30,31,32].

Thus, an adaptive integrated navigation algorithm (AMFKF) based on MFKF is proposed in this paper by combining the KF algorithm, the MFKF algorithm and the improved basis for judging the system state abnormality. An improved filter state anomaly judgment basis is designed by the algorithm in this paper to perform filter state switching, which is significantly better than the information fusion methods of KF and MFKF. The filter state anomaly judgment basis is often affected by the accuracy of the estimated value of the innovation covariance. Therefore, a recursive approach is used to continuously update the estimated value of the innovation covariance to improve the estimation accuracy of the innovation covariance in the improved filtering state anomaly judgment basis proposed in this paper. Moreover, in order to have stronger adaptability in practical engineering applications, the improved filter state abnormality judgment basis retains the adjustable reserve factor. The algorithm in this paper makes the timing of the introduction of the fading factor more reasonable by switching the filtering state to improve the filtering accuracy and enhance the adaptivity of the filter.

The rest of this paper is arranged as follows. In Section 2, INS/GNSS error model and KF algorithm are discussed in detail and the mathematical model is established. Section 3 analyzes the basic theories of MFKF. Then, an improved model anomaly judgment basis is designed. In Section 4, the experimental results of the algorithm in this paper are compared with the KF and MFKF in simulation and experiments. Finally, the conclusion based on the simulation and experiments is given in Section 5.

## 2. Integrated INS/GNSS Navigation System

### 2.1. Mathematical Error Model of INS/GNSS Integrated Navigation System

In this section, we shall establish a mathematical model of INS/GNSS loose integrated approach [33], in the east-north-up navigation reference frame,

(1) $$X(t)={\left[\begin{array}{ccccccccccccccc} \phi_{e} & \phi_{n} & \phi_{u} & \delta V_{e} & \delta V_{n} & \delta V_{u} & \delta L & \delta \lambda & \delta h & \varepsilon_{bx} & \varepsilon_{by} & \varepsilon_{bz} & \nabla_{ax} & \nabla_{ay} & \nabla_{az} \end{array}\right]}^{T}$$

where X(t) is the 15-dimensional state vector of the system. $\phi_{e}$, $\phi_{n}$, and $\phi_{u}$ represent the angle errors of the attitude in the direction of east, north, and up, respectively. $\delta V_{e}$, $\delta V_{n}$, and $\delta V_{u}$ are the three velocity errors in the direction of east, north, and up, respectively δL, δλ, and δh are the latitude, longitude, and altitude error respectively $\varepsilon_{bx}$, $\varepsilon_{by}$, and $\varepsilon_{bz}$ are gyro constant drifts, and $\nabla_{ax}$, $\nabla_{ay}$, and $\nabla_{az}$ are the random constant offset of the accelerometer.

Firstly, we analyze and model the inertial navigation system error. There are many kinds of errors in the inertial navigation system and their causes and correction methods are different. This paper only focuses on the errors caused by inertial devices, including platform angle error, velocity error, position error, gyroscope random drift, and accelerometer zero bias.

The differential expression for the platform angle error is:

(2) $$\overset{\cdot }{\phi^{n}}=\delta \omega_{ie}^{n}+\delta \omega_{en}^{n}-(\omega_{ie}^{n}+\omega_{en}^{n})\times \phi^{n}+\varepsilon^{n}$$

where $\phi^{n}={\left[\begin{array}{ccc} \phi_{e} & \phi_{n} & \phi_{u} \end{array}\right]}^{T}$, $\omega_{ie}^{n}$ is the vector projection of the angular velocity of the Earth’s rotation in the navigation coordinate system. $\omega_{en}^{n}$ is the projection of the implicated angular velocity vector from the rotation of the Earth in the navigation coordinate system. $\delta \omega_{ie}^{n}$ is the earth rotation error’s projection and $\delta \omega_{en}^{n}$ is geographic coordinate error’s projection, which is caused by the error of latitude and speed. $\varepsilon^{n}$ is the gyro constant error’s projection of INS in the navigation coordinate system.

From the above equation, the angle error of the attitude equation can be further derived as:

(3) $$\begin{array}{l} \overset{\cdot }{\phi_{e}}=\;(\omega_{ie}\sin L+\frac{V_{e}}{R_{n}+h}\tan L)\phi_{n}-(\omega_{ie}\cos L+\frac{V_{e}}{R_{n}+h})\phi_{u}-\frac{\delta V_{n}}{R_{m}+h}\\ +\frac{V_{n}}{{\left(R_{m}+h\right)}^{2}}\delta h+\varepsilon_{e} \end{array}$$

(4) $$\begin{array}{l} \overset{\cdot }{\phi_{n}}=-(\omega_{ie}\sin L+\frac{V_{e}}{R_{n}+h}\tan L)\phi_{e}-\frac{V_{n}}{R_{m}+h}\phi_{u}-\omega_{ie}\sin L\delta L+\frac{\delta V_{e}}{R_{n}+h}\\ -\frac{V_{e}}{{\left(R_{n}+h\right)}^{2}}\delta h+\varepsilon_{n} \end{array}$$

(5) $$\begin{array}{l} \overset{\cdot }{\phi_{u}}=(\omega_{ie}\cos L+\frac{V_{e}}{R_{n}+h})\phi_{e}+\frac{V_{n}}{R_{m}+h}\phi_{n}+(\omega_{ie}\cos L+\frac{V_{e}}{R_{n}+h}\sec^{2}L)\delta h\\ +\frac{\delta V_{e}}{R_{n}+h}\tan L+\varepsilon_{u} \end{array}$$

where, $V_{e}$ and $V_{n}$ are the velocity from directions of east and north obtained by inertial navigation system, respectively. $R_{n}$ and $R_{m}$ are the major axis and short axis of earth. h the local height of the carrier. $\varepsilon_{e}$, $\varepsilon_{n}$, and $\varepsilon_{u}$ represent the error of the gyro in the direction of east, north, and up, respectively.

According to the specific force equation:

(6) $$f^{n}=\overset{\cdot }{V^{n}}+(2\omega_{ie}+w_{en})\times V^{n}-g^{n}$$

where $f^{n}$ is the projection of the specific force value measured by the accelerometer in the navigation coordinate system; $V^{n}$ is the velocity from direction of east, north, and up; $g^{n}$ is the projection of local gravity in the navigation coordinate system.

The velocity error equation is obtained as:

(7) $$\delta \overset{\cdot }{V^{n}}=f^{n}\times \phi^{n}+\nabla^{n}-(2\delta \omega_{ie}+\delta \omega_{en})\times V^{n}-(2\delta \omega_{ie}+\omega_{en})\times \delta V^{n}$$

where $\nabla^{n}$ is the accelerometer error’s projection of INS in navigation coordinate system.

Then states of velocity error can be established as:

(8) $$\begin{array}{l} \delta \overset{\cdot }{V_{e}}=\;(\frac{V_{n}}{R_{n}+h}\tan L-\frac{V_{u}}{R_{n}+h})\delta V_{e}+(2\omega_{ie}\sin L+\frac{V_{e}}{R_{n}+h}\tan L)\delta V_{n}\\ +(2\omega_{ie}V_{n}\cos L+\frac{V_{e}V_{n}}{R_{n}+h}\sec^{2}L+2\omega_{ie}V_{u}\sin L)\delta L\\ -(2\omega_{ie}\cos L+\frac{V_{e}}{R_{n}+h})\delta V_{u}+\phi_{u}f_{n}-\phi_{u}f_{u}+\nabla_{e} \end{array}$$

(9) $$\begin{array}{l} \delta \overset{\cdot }{V_{n}}=-(2\omega_{ie}\sin L+\frac{2V_{e}}{R_{n}+h}\tan L)\delta V_{e}-\frac{V_{u}}{R_{m}+h}\delta V_{n}-\frac{V_{n}}{R_{m}+h}\delta V_{u}\\ -(2\omega_{ie}V_{e}\cos L+\frac{V_{e}^{2}}{R_{n}+h}\sec^{2}L)\delta L-\phi_{u}f_{e}+\phi_{e}f_{u}+\nabla_{n} \end{array}$$

(10) $$\begin{array}{l} \delta \overset{\cdot }{V_{u}}=2(\omega_{ie}\cos L+\frac{V_{e}}{R_{n}+h})\delta V_{e}+\frac{2V_{n}}{R_{m}+h}\delta V_{n}-2\omega_{ie}V_{e}\sin L\delta L+\phi_{n}f_{e}\\ -\phi_{e}f_{n}+\nabla_{u} \end{array}$$

where $f_{e}$, $f_{n}$, and $f_{u}$ represent the specific force in the direction of east, north, and up, respectively. $\nabla_{e}$, $\nabla_{n}$, and $\nabla_{u}$ represent the error of the accelerometer in the direction of east, north, and up, respectively. 

After obtaining the error of velocity, the position error can be figured out. Then states of position error can be established as:

(11) $$\delta \overset{\cdot }{L}=\frac{\delta V_{n}}{R_{m}+h}-\frac{V_{n}}{{\left(R_{m}+h\right)}^{2}}\delta h$$

(12) $$\delta \overset{\cdot }{\lambda }=\frac{\delta V_{e}}{R_{n}+h}\sec L+\frac{V_{e}}{R_{n}+h}\delta L\sec L\tan L-\frac{V_{e}\sec L}{{\left(R_{n}+h\right)}^{2}}\delta h$$

(13) $$\delta \overset{\cdot }{h}=\delta V_{u}$$

The constant error and noise in the navigation coordinate system are:

(14) $$\varepsilon^{n}=C_{b}^{n}\varepsilon^{b},\nabla^{n}=C_{b}^{n}\nabla^{b}$$

(15) $$\omega_{g}^{n}=C_{b}^{n}\omega_{g},\omega_{a}^{n}=C_{b}^{n}\omega_{a}$$

where $C_{b}^{n}$ is the coordinate transformation matrix from the body frame to the navigation frame. $\varepsilon^{b}$ is gyro constant drift, $\nabla^{b}$ is the offset of the accelerometer, $\omega_{g}$ is the gyro noise, and $\omega_{a}$ is the accelerometer noise.

Based on the established inertial navigation error model, the equations of state can be given by Equation (16):

(16) $$\overset{\cdot }{X}(t)=F(t)\cdot X(t)+G(t)\cdot W(t)$$

where F(t) is the state transition matrix; G(t) is the matrix of noise driving, determined by Equation (15); and W(t) is the system state noise vector.

(17) $$F(t)=\left[\begin{array}{ccccc} M_{aa} & M_{av} & M_{ap} & C_{b}^{n} & 0_{3\times 3}\\ M_{va} & M_{vv} & M_{vp} & 0_{3\times 3} & C_{b}^{n}\\ 0_{3\times 3} & M_{pv} & M_{pp} & 0_{3\times 3} & 0_{3\times 3}\\ & & 0_{6\times 15} & & \end{array}\right]$$

where $M_{aa}$, $M_{av}$, $M_{ap}$, $M_{va}$, $M_{vv}$, $M_{vp}$, $M_{pv}$, $M_{pp}$ can be represented individually as:

(18) $$M_{aa}=\left[\begin{array}{ccc} 0 & \omega_{ie}\sin L+\frac{V_{e}\tan L}{R_{n}+h} & -(\omega_{ie}\cos L+\frac{V_{e}}{R_{n}+h})\\ -(\omega_{ie}\sin L+\frac{V_{e}\tan L}{R_{n}+h}) & 0 & -\frac{V_{n}}{R_{m}+h}\\ \omega_{ie}\cos L+\frac{V_{e}}{R_{n}+h} & \frac{V_{n}}{R_{m}+h} & 0 \end{array}\right]$$

(19) $$M_{av}=\left[\begin{array}{ccc} 0 & \frac{1}{R_{m}+h} & 0\\ \frac{1}{R_{n}+h} & 0 & -\frac{V_{n}}{R_{m}+h}\\ \frac{\tan L}{R_{n}+h} & 0 & 0 \end{array}\right]$$

(20) $$M_{ap}=\left[\begin{array}{ccc} 0 & 0 & 0\\ -\omega_{ie}\sin L & 0 & 0\\ \omega_{ie}\cos L+\frac{V_{e}}{R_{n}+h}\sec^{2}L & 0 & 0 \end{array}\right]$$

(21) $$M_{va}=\left[\begin{array}{ccc} 0 & -f_{u} & f_{n}\\ f_{u} & 0 & f_{e}\\ -f_{n} & f_{e} & 0 \end{array}\right]$$

(22) $$M_{vv}=\left[\begin{array}{ccc} \frac{V_{n}\tan L}{R_{n}+h}-\frac{V_{u}}{R_{n}+h} & 2\omega_{ie}\sin L+\frac{V_{e}\tan L}{R_{n}+h} & 2\omega_{ie}\cos L+\frac{V_{e}}{R_{n}+h}\\ -2\omega_{ie}\sin L+\frac{V_{e}\tan L}{R_{n}+h} & -\frac{V_{u}}{R_{m}+h} & -\frac{V_{n}}{R_{m}+h}\\ 2(\omega_{ie}\cos L+\frac{V_{e}}{R_{n}+h}) & \frac{2V_{n}}{R_{m}+h} & 0 \end{array}\right]$$

(23) $$M_{vp}=\left[\begin{array}{ccc} 2\omega_{ie}\cos LV_{n}+\frac{V_{e}V_{n}}{R_{n}+h}\sec^{2}L+2\omega_{ie}\sin LV_{u} & 0 & 0\\ -2\omega_{ie}\cos LV_{e}-\frac{V_{e}^{2}}{R_{n}+h}\sec^{2}L & 0 & 0\\ -2\omega_{ie}\sin LV_{e} & 0 & 0 \end{array}\right]$$

(24) $$M_{pv}=\left[\begin{array}{ccc} 0 & -\frac{1}{R_{m}+h} & 0\\ \frac{\sec L}{R_{n}+h} & 0 & 0\\ 0 & 0 & 1 \end{array}\right]$$

(25) $$M_{pp}=\left[\begin{array}{ccc} 0 & 0 & 0\\ \frac{V_{e}\sec L\tan L}{R_{n}+h} & 0 & 0\\ 0 & 0 & 0 \end{array}\right]$$

(26) $$G(t)=\left[\begin{array}{cc} C_{b}^{n} & 0_{3\times 3}\\ 0_{3\times 3} & C_{b}^{n}\\ 0_{9\times 3} & 0_{9\times 3} \end{array}\right]$$

(27) $$W(t)=\left[\begin{array}{cccccc} w_{gx} & w_{gy} & w_{gz} & w_{ax} & w_{ay} & w_{az} \end{array}\right]$$

$\omega_{gx}$, $\omega_{gy}$, and $\omega_{gz}$ are the Gaussian white noise of the three axis systems of the gyro and $\omega_{ax}$, $\omega_{ay}$, and $\omega_{az}$ are the Gaussian white noise of the three axis systems of the accelerometer.

The current position information of the system is obtained by the GNSS receiver, and then the difference between the position output by INS solution and the position output by GNSS is used to obtain the system observation equation:

(28) $$Z(t)=\left[P_{INS}-P_{GNSS}\right]=\left[\begin{array}{c} L_{INS}-L_{GNSS}\\ \lambda_{INS}-\lambda_{GNSS}\\ h_{INS}-h_{GNSS} \end{array}\right]=H(t)X(t)+V(t)$$

where the position information output from INS consists of the actual position and the solution error, which can be written as the following equation:

(29) $$\begin{array}{l} L_{INS}=L+\delta L_{INS}\\ \lambda_{INS}=\lambda +\delta \lambda_{INS}\\ h_{INS}=h+\delta h_{INS} \end{array}$$

The GNSS position information consists of the actual position and the position error from the GNSS receiver, which can be written as the following equation:

(30) $$\begin{array}{l} L_{GNSS}=L+\delta L_{GNSS}\\ \lambda_{GNSS}=\lambda +\delta \lambda_{GNSS}\\ h_{GNSS}=h+\delta h_{GNSS} \end{array}$$

H(t) can be represented as:

(31) $$H(t)=\left[\begin{array}{ccc} 0_{3\times 6} & I_{3\times 3} & 0_{3\times 6} \end{array}\right]$$

V(t) can be represented as:

(32) $$V(t)={\left[\begin{array}{ccc} \eta_{L} & \eta_{\lambda } & \eta_{h} \end{array}\right]}^{T}$$

where $\eta_{L}$, $\eta_{\lambda }$, and $\eta_{h}$ represent the errors with noise when the GNSS receiver acquires the current latitude, longitude, and altitude, respectively, and all are Gaussian white noise.

### 2.2. KF Algorithm

The equation of state and the observation equation of the discrete system are:

(33) $$X_{k}=\Phi_{k/k-1}X_{k-1}+\Gamma_{k-1}W_{k-1}$$

(34) $$Z_{k}=H_{k}X_{k}+V_{k}$$

where $X_{k}$ is the state vector; $Z_{k}$ is the system observation; $\Phi_{k/k-1}$ is the state transition matrix; $\Gamma_{k-1}$ is the matrix of noise; $H_{k}$ is the observation matrix; $W_{k-1}$ is the system noise vector; and $V_{k}$ is the observation noise vector of the systematic. Meanwhile, $W_{k}$ and $V_{k}$ are independent of each other and both belong to zero-mean white noise.

The KF equation is as follows.

State one-step predicting equation:

(35) $${\overset{\wedge }{X}}_{k/k-1}=\Phi_{k/k-1}{\overset{\wedge }{X}}_{k-1}$$

Error variance matrix of one-step predicting:

(36) $$P_{k/k-1}=\Phi_{k/k-1}P_{k-1}\Phi_{k/k-1}^{T}+Q_{k}$$

Kalman gain matrix:

(37) $$K_{k}=P_{k/k-1}H^{T}{\left[H_{k}P_{k/k-1}H_{k}^{T}+R_{k}\right]}^{-1}$$

Innovation:

(38) $$e_{k}=Z_{k}-H_{k}{\overset{\wedge }{X}}_{k/k-1}$$

State estimation equation:

(39) $${\overset{\wedge }{X}}_{k}={\overset{\wedge }{X}}_{k/k-1}+K_{k}e_{k}$$

Estimation error variance matrix:

(40) $$P_{k}=(I-K_{k}H_{k})P_{k/k-1}$$

## 3. The Proposed Method with Both Adaptivity and Robustness

### 3.1. Basic Theories of Fading Filtering

The basic idea of the fading filter is to increase the error variance matrix of the one-step predicting of the state estimate by an appropriate fading factor, then adjusts the filter gain matrix, thus increasing the weight of the innovation and suppressing the filter divergence. A typical fading filter differs from the KF by introducing a fading factor λ into the filter Equation (36). According to the theory, the error variance matrix of one-step predicting of the fading filter can be given by (41).

(41) $$P_{k/k-1}=\lambda \Phi_{k/k-1}P_{k-1}\Phi_{k/k-1}^{T}+Q_{k}$$

Of these, the selection of the fading factor *λ* is the key of fading filtering.

In the traditional single fading factor Kalman filter, the fading factor is selected as follows:

(42) $$\lambda_{k}=\max\left(1,\frac{trace(N_{k})}{trace\left(M_{k}\right)}\right)$$

where

(43) $$N_{k}={\overset{\wedge }{C}}_{k}-H_{k}Q_{k}H_{k}^{T}-R_{k}$$

(44) $$M_{k}=H_{k}\Phi_{k/k-1}P_{k-1}\Phi_{k/k-1}^{T}H_{k}^{T}$$

${\overset{\wedge }{C}}_{k}$ is the estimate of the innovation sequence $e_{k}$, usually obtained as follows [34]:

(45) $${\overset{\wedge }{C}}_{k}=\frac{1}{k}\sum_{i=1}^{k}e_{i}e_{i}^{T}$$

The innovation sequence covariance matrix can be obtained from Equation (38) as

(46) $$C_{k}=H_{k}P_{k/k-1}H_{k}^{T}+R_{k}$$

An important feature of linear optimal Kalman filtering is that the innovation sequence $e_{k}$ is a white noise sequence when the gain matrix $K_{k}$ is optimal. Thus:

(47) $$\begin{array}{l} E[e_{k+j}e_{k}^{T}]=H_{k+j}\Phi_{k+j-1,k+j-2}\cdot \\ {}[I-K_{k+j-1}H_{k+j-1}]\Phi_{k+1,k}[I-K_{k+1}H_{k+1}]\cdot \\ \Phi_{k,k-1}[P_{k/k-1}H_{k}^{T}-K_{k}C_{k}],\forall j=1,2,3,\cdots \end{array}$$

Substituting Equations (37) and (46) into Equation (47), then

(48) $$E[e_{k+j}e_{k}^{T}]=P_{k/k-1}H_{k}^{T}-K_{k}C_{k}=0$$

That is, the innovation sequence satisfies Equation (48) under the Kalman filtering optimality condition. However, in the actual case, the actual innovation covariance matrix is not the same as the calculated theoretical value due to the inaccuracy of the system model or noise statistical properties, resulting in Equation (48) not being valid. The basic principle of the fading filter is that by selecting an appropriate fading factor λ, the gain matrix $K_{k}$ is adjusted in real time to force the innovation sequences to be orthogonal to each other so that Equation (48) holds, thus achieving a suppression of filter divergence.

### 3.2. Multiple Fading Factors Kalman Filter

The diagonal elements in the error covariance matrix $P_{k/k-1}$ represent the estimation accuracy of the corresponding state variables, while the single fading factor cannot fully utilize the adjustment performance due to the same adjustment weights for each state channel of $P_{k/k-1}$. The multiple fading factors assign different fading weights to each state channel, which can better adjust the gain matrix adaptively and thus enhance the stability of filtering when the system model is mismatched or the noise is not matched.

Error variance matrix of one-step predicting:

(49) $$P_{k/k-1}=\left[\begin{array}{ccc} p(1,1) & & \\ & \ddots & \\ & & p(n,n) \end{array}\right]$$

where p(i,i) denotes the estimation accuracy of the *i*th state variable in the state vector.

The multiple fading factors matrix is described as follows.

(50) $$\Lambda =\left[\begin{array}{ccc} \lambda (1,1) & & \\ & \ddots & \\ & & \lambda (n,n) \end{array}\right]$$

where λ(i,i) denotes the fading factor corresponding to the *i*th state variable assignment and satisfies:

(51) λ(i,i)=c(i)⋅λ(k)

where λ(i,i)≥1, λ(k) is the scalar fading factor derived from Equation (42) and c(i) is the weight factor assigned by λ(i,i) and satisfies:

(52) $$\{\begin{array}{c} \bar{c}\left(i\right)=p(i,i)/(trace(P_{k/k-1})/n)\\ c\left(i\right)=\max(1,\bar{c}(i)) \end{array}$$

### 3.3. An Improved Model Anomaly Judgment Basis

In engineering applications, according to the theory of KF orthogonality, when the system model is inaccurate or mismatched, it will cause the KF to gradually diverge and the innovation sequence will increase significantly; therefore, it is usually based on the innovation covariance to determine whether the filter is normal.

The traditional basis for judging system model state anomalies [35] is:

(53) $$e_{k}^{T}e_{k}>\gamma tr(E\left[e_{k}e_{k}^{T}\right])$$

where γ is an adjustable empirical reserve factor more than 1. Ideally, the calculated value of the actual innovation covariance on the left side of the equation is equal to the expected value of the innovation covariance on the right side, reflecting the degree of the deviation of the actual error from the theoretical error. However, replacing the estimate of the new interest covariance with $e_{k}^{T}e_{k}$ in the traditional way is less reasonable and prone to misclassification.

In this paper, the actual innovation covariance can be estimate by Equation (54)

(54) $${\overset{\wedge }{C}}_{k}=\{\begin{array}{c} e_{1}e_{1}^{T},k=0\\ \frac{\rho {\overset{\wedge }{C}}_{k-1}+e_{k}e_{k}^{T}}{1+\rho },k>0 \end{array}$$

where ρ is the forgetting factor, 0<ρ≤1, which is usually taken as = 0.95. This method increases the weights of the innovation sequence and improves the accuracy of the estimated value of the innovation sequence covariance and, as a recursive approach, is simpler to implement, uses less data storage, and is more suitable for use in combinatorial navigation systems with high dimensionality.

From Equation (34), the observation noise $V_{k}$ satisfies the following properties:

(55) $$\{\begin{array}{c} E[V_{k}]=0\\ Cov[V_{k}V_{k}]=R_{k} \end{array}$$

The Equation (37) can be written in the following form:

(56) $$K_{k}=P_{k/k-1}H^{T}C_{k}^{-1}$$

where $C_{k}$ is the theoretical value of the innovation sequence covariance.

(57) $$C_{k}=H_{k}P_{k/k-1}H_{k}^{T}+R_{k}$$

Thus, theoretically, in the filter-optimal case, the innovation sequence covariance is

(58) $$E\left[e_{k}e_{k}^{T}\right]=H_{k}P_{k/k-1}H_{k}^{T}+R_{k}$$

Substituting Equations (54) and (58) into Equation (53) and taking traces on both sides of the equation, the new system state abnormality judgment basis can then be given by:

(59) $$\mathrm{tr}\left({\overset{\wedge }{C}}_{k}\right)=\gamma tr\left(H_{k}P_{k/k-1}H_{k}^{T}+R_{k}\right)$$

From the above Equation (2), it can be seen that the new system anomaly judgment basis designed in this paper is significantly better than the traditional judgment basis and is innovative. Firstly, it is considered that the accuracy of the innovative covariance estimation value often affects the judgment basis of filter state anomaly. Therefore, in the improved filter state anomaly judgment basis proposed in this paper, the recursive method is used to continuously update the innovation covariance estimation value, which improves the estimation accuracy of the innovation covariance, and such a method makes the judgment more rigorous and precise. In addition, the improved filter state anomaly judgment basis retains the adjustable reserve factor, which has stronger adaptability in practical engineering applications.

Thus, an adaptive integrated navigation algorithm based on MFKF (AMFKF) is proposed in this paper by combining the KF algorithm, the MFKF algorithm, and the improved basis for judging the system state abnormality. If the system model is judged to be abnormal, it is switched to the MFKF algorithm; otherwise, the optimal estimation is performed according to the KF. In summary, the algorithm in this paper designs an improved basis for judging system state abnormality based on MFKF, which makes the timing of fading factor introduction more reasonable and the information fusion effect better than the KF and the MFKF algorithms through the switching of filtering states.

**Figure 1:**
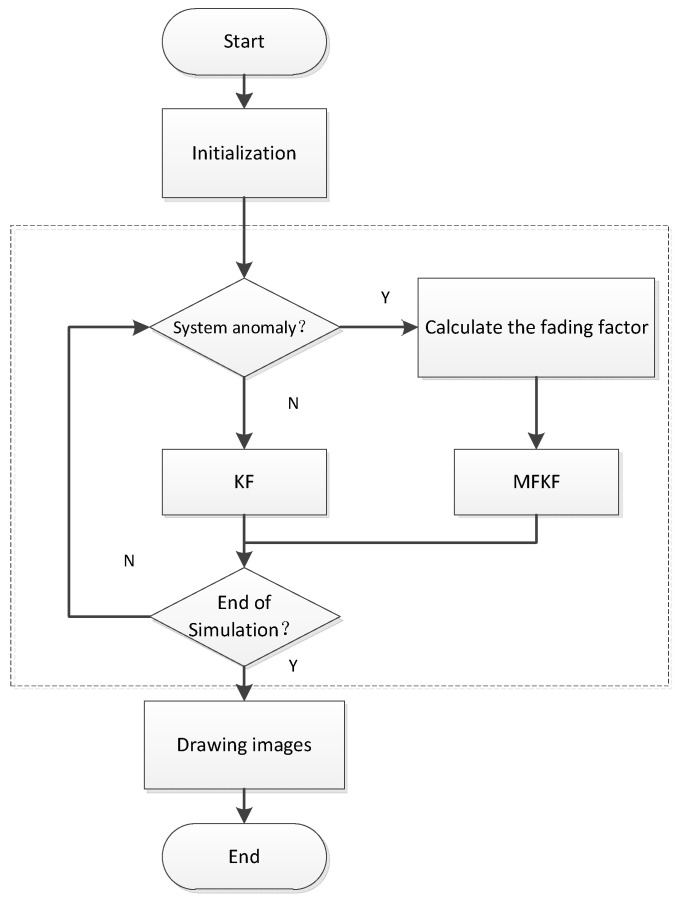
The flow chart of the integrated navigation algorithm based on MFKF.

The basic flow chart of the algorithm in this paper is shown in Figure 1.

## 4. Experiments and Discussion

### 4.1. Simulation Experiments

In order to verify the performance of the algorithm proposed in the article for the integrated INS/GNSS navigation system, a simulation study was be conducted, and the results will be analyzed in this paper.

The initial position of the vehicle was assumed to be 120.710° E, 36.099° N, and 38 m in height. The simulation parameters were set as follows: IMU technical parameters are shown in Table 1, GNSS sampling frequency is 1 Hz, and the initial error angle of the platform is 0.1°. The initial velocity error is 0.1 m/s and the initial position error is 1 m.

**Figure 2:**
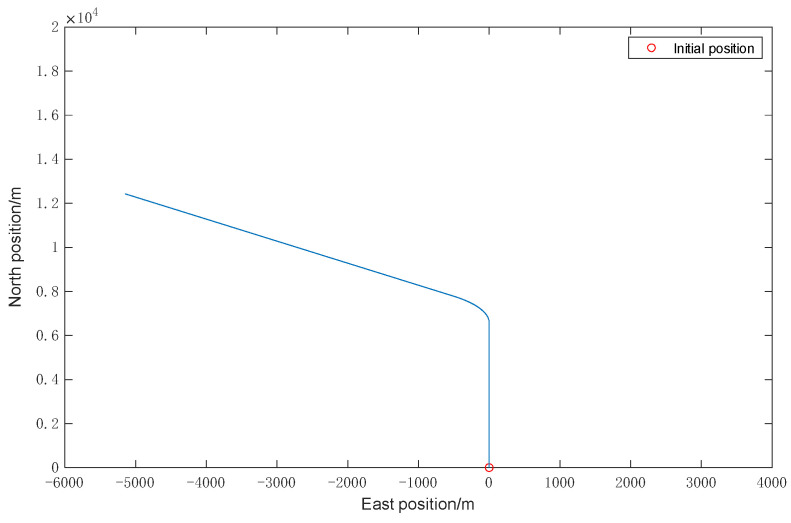
Vehicle trajectory.

**Figure 3:**
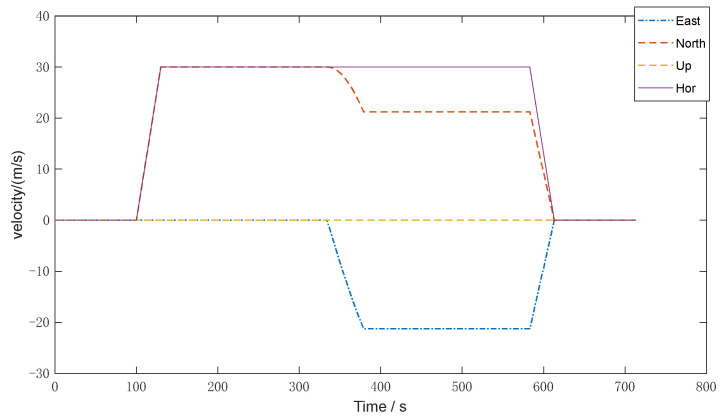
Vehicle velocity.

To simulate the running status of acceleration and deceleration, straight ahead, turning, and so on, the trajectory and velocity of the vehicle set are shown in Figure 2 and Figure 3, respectively.

In order to verify the performance of the improved algorithm, KF, MFKF, and the algorithm of this paper are selected for comparison and simulation. By setting the simulation process with an accurate system model as well as the noise statistical characteristics, in the period of 400~700 s, the system noise $Q_{k}$ abruptly changed to 200 times the original, simulating the situation of system noise mismatch during the vehicle driving.

**Figure 4:**
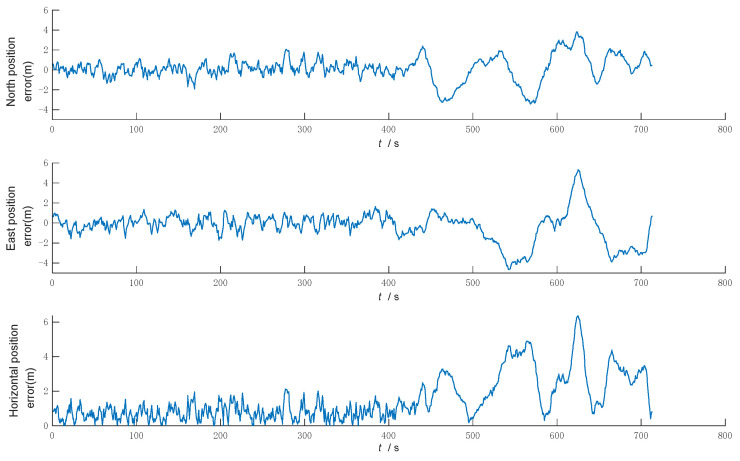
Error of Kalman filtering.

**Figure 5:**
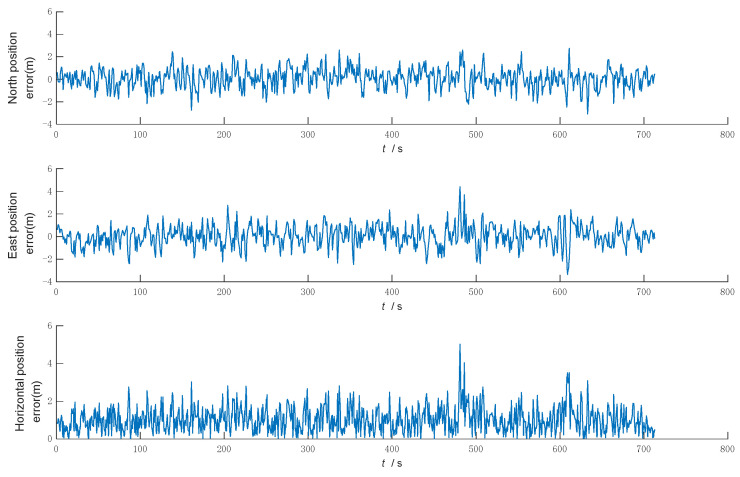
Error of multiple fading factors Kalman filtering.

**Figure 6:**
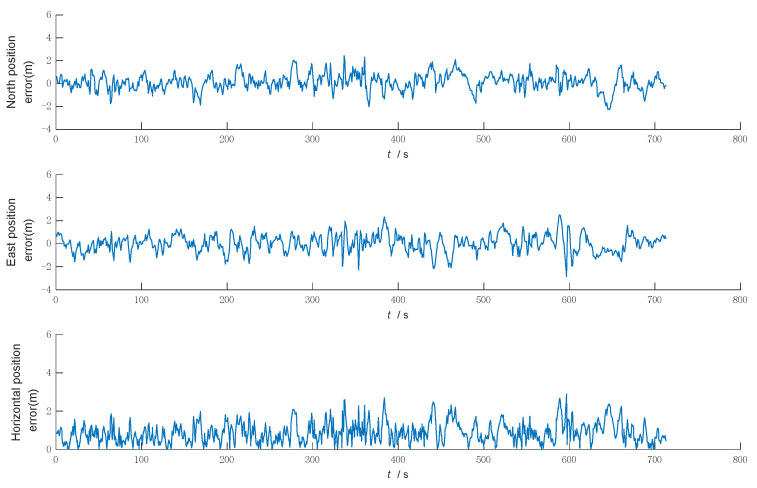
Error of the filtering algorithm in this paper.

Under the same conditions, the above three filtering algorithms are simulated and compared, respectively, and the simulation results output the northward, eastward, and horizontal position errors, as shown in Figure 4, Figure 5 and Figure 6.

Figure 4 shows that the KF has good filtering accuracy and stability when the system model matches and the noise statistics are known. However, when there is a sudden change in the system noise, the system noise statistic characteristics no longer match, the filtering accuracy becomes poor, and the filtering gradually diverges and loses its filtering effect.

Figure 5 shows that MFKF is effective in controlling the filtering dispersion by increasing the weight of the newly observed data with the fading factor when the statistical characteristics of the noise do not match and the filtering dispersion occurs. However, in the whole simulation process, when the filter divergence control is not needed, the overuse of fading factor leads to the reduction of the accuracy and even to adverse effects, such as sudden changes in the filtering accuracy.

Figure 6 shows that the filtering algorithm in this paper uses the KF when the filter is normal, and when the filter is judged to be divergent, the system switches to MFKF, which can effectively control the trend of filter divergence by adaptive adjustment based on the improved filter anomaly judgment.

In order to better illustrate the effectiveness of the algorithms in this paper, the statistical results of the error mean and standard deviation of the three filtering algorithms are shown in Table 2.

As shown in Table 2, since the KF cannot suppress the filtering divergence, the mean error and the standard deviation of the error for each state are the largest. Meanwhile, the mean error and standard deviation of the error in each state of the algorithm of this paper are minimum, in which the mean error of the horizontal position is reduced by 40.9% and 13.4% compared with the KF and the MFKF, respectively; the standard deviation of the horizontal position error is reduced by 59.8% and 13.4% compared with the KF and the MFKF, respectively. Therefore, it indicates that the algorithm of this paper has improved the localization accuracy and stability compared with MFKF and has a strong filtering performance.

### 4.2. Actual Data Verification

**Figure 7:**
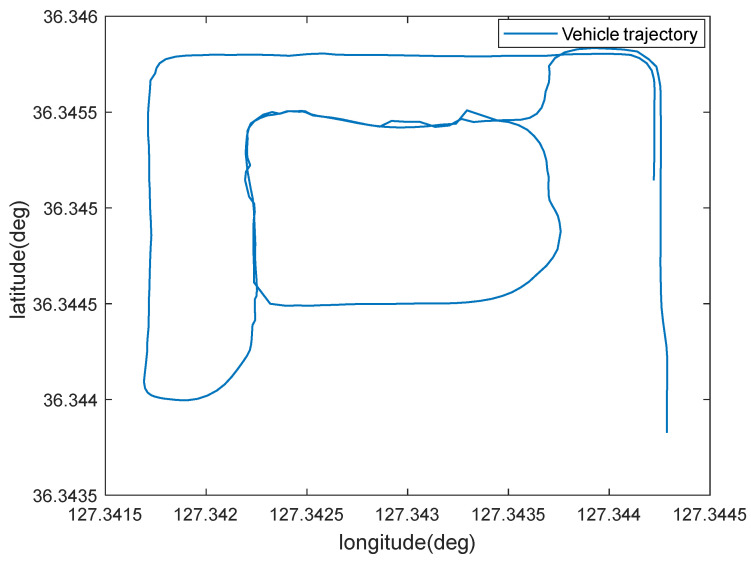
Trajectory of the vehicle.

**Figure 8:**
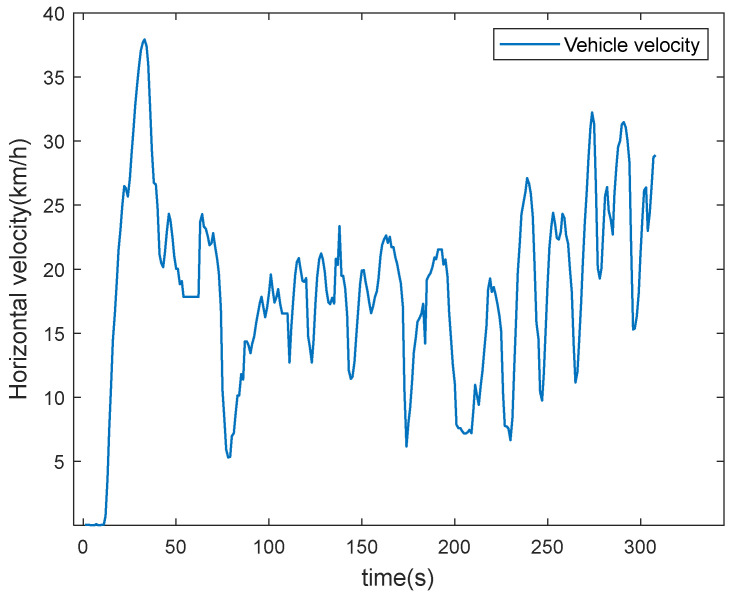
Velocity of the vehicle.

In order to further verify the practicality and feasibility of the algorithm in this paper, experimental validation is carried out using real vehicle data [36]. The MEMS device is XSENS MTi-300, which has superior performance, and the technical parameters are shown in Table 3; the GNSS receiver is a U-Blox EVK-7P with a sampling frequency of 5 Hz. The trajectory and velocity of vehicle are shown in Figure 7 and Figure 8.

From Figure 7 and Figure 8, it can be seen that the vehicle has obvious acceleration and deceleration and significant turning movements during the whole driving process.

**Figure 9:**
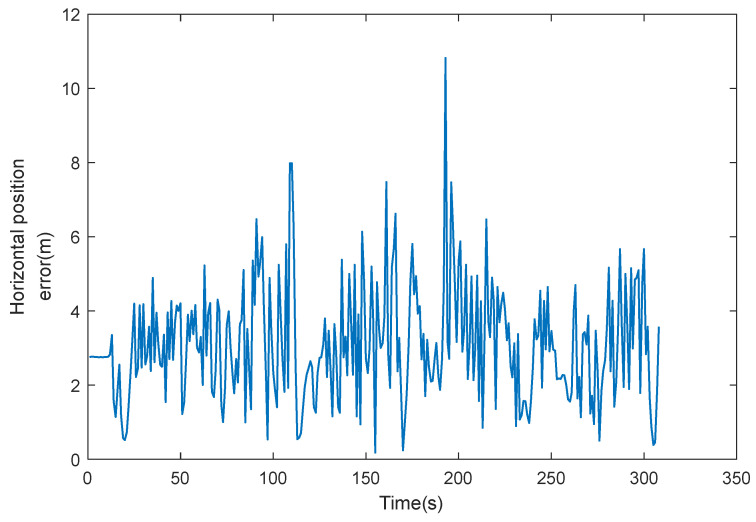
GNSS positioning error.

The error curves of GNSS positioning are shown in Figure 9, and the positioning error statistics are shown in Table 4.

It is shown in Figure 9 and Table 4 that GNSS navigation positioning has unstable errors, which is due to the fact that GNSS navigation positioning results are influenced by satellite signals, and when the vehicle is located between buildings or in areas with tree shading, multipath effects will occur, resulting in significant errors during that time period, so GNSS navigation alone is less reliable.

Since the performance of inertial navigation sensors is affected by external environmental conditions, in this paper, the system noise data is artificially modified in the filter to simulate the effect of system noise mismatch or interference from the external environment in order to verify the feasibility and effectiveness of the algorithm proposed in this paper. That is, assuming that in the period from 200 s to 300 s, the system noise is affected by the outside world and the real value of the system noise becomes 10 times the statistical characteristics of the system noise, while the system noise is still considered constant in the filter.

**Figure 10:**
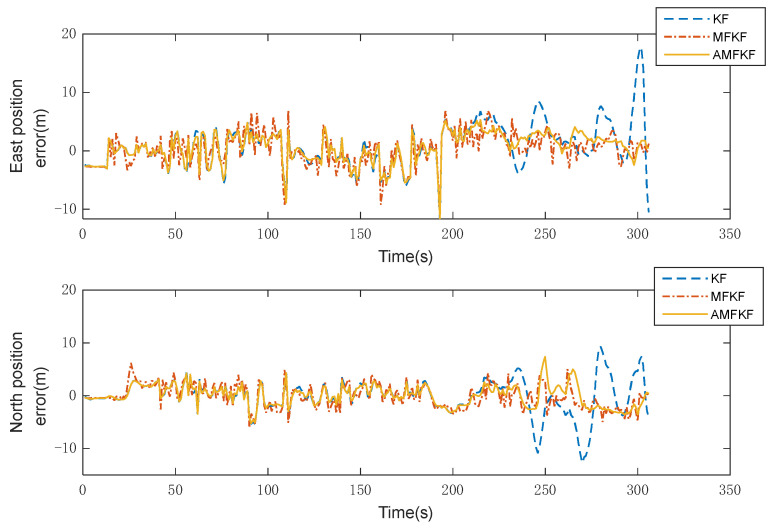
Eastward and northward error.

**Figure 11:**
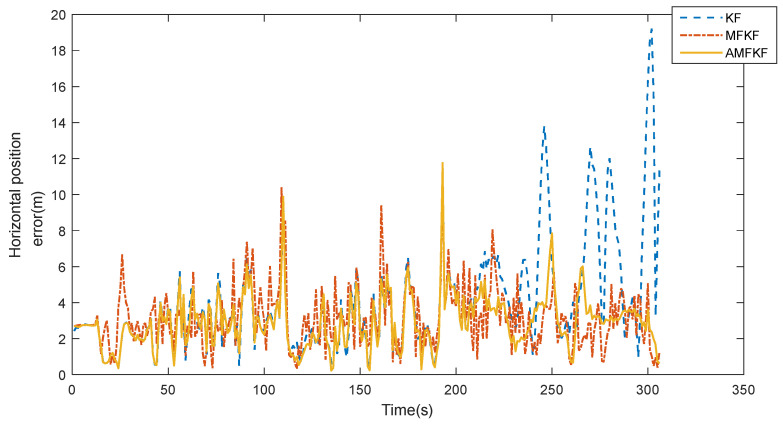
Horizontal position error.

Experimental results: The comparison curves of the eastward and northward error of the integrated INS/GNSS navigation position are shown in Figure 10, and the comparison curves of the horizontal position error are shown in Figure 11.

Experimental results: Figure 10 and Figure 11 show the error curves of KF, MFKF, and the proposed algorithm (AMFKF). The positioning accuracy of MFKF is relatively low due to the overuse of the fading factor when the system noise is not seriously disturbed. Combined with the principal analysis of the MFKF in Section 3.2 of the article, it is clear that the basic idea of the MFKF is to increase the error variance matrix of the one-step prediction of the state estimation by appropriate fading factor, and then adjust the filter gain matrix so as to increase the weight of the innovation and suppress the filter divergence. However, when the filter state is normal, the fading factor is used to excessively increase the weight of the innovation at any time, resulting in a mismatch between the noise information of the filter and the true, which leads to a decrease in filtering accuracy.

In the time period of 200~300 s, KF has an obvious tendency to diverge and gradually lose its filtering performance due to the serious mismatch of system noise. However, the AMFKF can continuously switch the filtering state through the improved filtering state judgment basis in this paper. When the system determines that the filter tends to diverge, it switches to the MFKF to suppress the filter divergence, and when the filter state returns to normal, it switches to the KF. Therefore, AMFKF performs better than KF and MFKF in terms of filtering stability and filtering accuracy.

Combined with Table 5, it can be seen that the average positioning accuracy of AMFKF is improved by 26.52% and 7.48% in the mean value of the horizontal position error, and the standard deviation of the errors is reduced by 51.20%, 12.16%, respectively, compared with the KF and MFKF, due to the more reasonable timing of the introduction of the fading factor.

**Figure 12:**
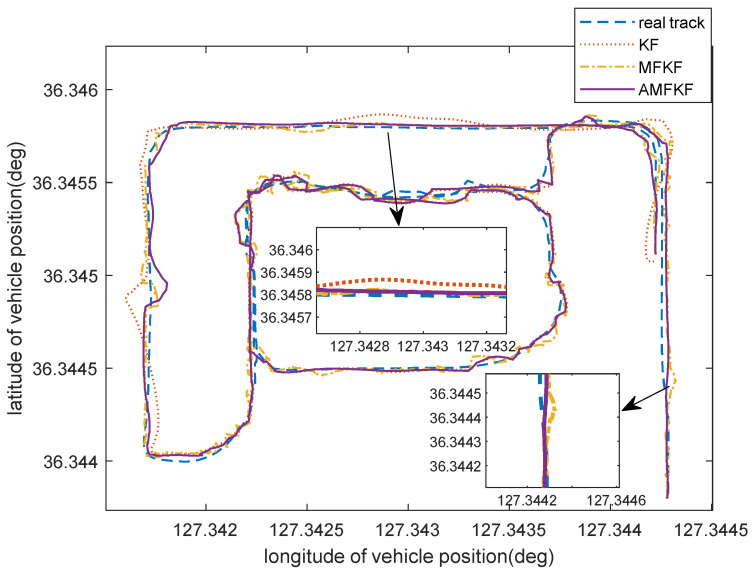
Integrated INS/GNSS navigation track with different algorithms.

Furthermore, the integrated INS/GNSS navigation track is output in order to verify the positioning performance of the algorithm in this paper, as shown in Figure 12.

Figure 12 shows the localization performance of KF algorithm, MFKF algorithm, and the algorithm in this paper (AMFKF). In general, there is no significant difference between the localization track of KF and AMFKF; however, MFKF suffers from a decrease in localization accuracy due to the overuse of the fading factor. It can also be seen from the Figure 12 that the MKFK localization trajectory performs worse than the KF and the AMFKF during the time period when there are no anomalies in the system.

In the section where the system noise mismatch occurs, the localization track of the KF algorithm has a significant tendency to drift relative to the true reference track, while the algorithm in this paper and the MFKF is estimated to be closer to the true track due to the suppression of the filtering divergence. Hence, the experimental results show that the algorithm in this paper can suppress the filtering divergence and improve the localization accuracy, which has certain feasibility and effectiveness.

### 4.3. Discussion

Based on the MFKF, a state-switching adaptive filter is proposed to solve the problem that the filtering accuracy is reduced due to the overuse of fading factor in the MFKF. It can be seen from the simulation results that the algorithm in this paper can suppress the divergence of filtering and improve the filtering accuracy when the system noise changes.

In practical applications, the system noise is not stable and constant, especially when it is affected by a harsh environment. Therefore, in the real vehicle data experiments, we simulated the condition when the system noise was set to be 10 times greater during the specified interval than the others. It can be seen from Figure 10 and Figure 11 that our proposed algorithm has the smallest estimation error, and Figure 12 also visually illustrates that our proposed algorithm has more accurate estimation results. The MFKF does not perform as well as our proposed algorithm because it cannot introduce the fading factor adaptively, and the overuse of the fading factor will reduce the filtering accuracy when the filtering state is normal.

In the future, we intend to add the adaptive adjustment of the measurement noise covariance. In practical applications, the complexity of the environment in which the moving target is located can lead to time-varying measurement noise covariance, which in turn leads to increased filtering errors. We will adaptively update the measurement noise covariance to adapt to environmental changes and improve the filtering accuracy. Therefore, a filtering algorithm with measurement noise covariance adaptive update will further improve the estimation accuracy.

## 5. Conclusions

An integrated navigation algorithm based on a multiple fading factors Kalman filter is proposed in this paper, taking an INS/GNSS integrated navigation system as an example, which overcomes the divergence problem of KF in the case of noise–statistical characteristic mismatch and avoids the problem of filtering accuracy degradation caused by the overuse of MFKF. The algorithm performs filter state switching based on the improved system anomaly judgment basis, which makes the timing of the introduction of the fading factor more reasonable and the algorithm more adaptive.

Different from the traditional basis of filter abnormality judgment, in the improved basis of filter state abnormality judgment proposed in this paper, the recursive method is used to continuously update the innovation covariance estimates, which improves the estimation accuracy of the innovation covariance and makes the judgment more rigorous and precise. Thus, the method of retaining the adjustable reserve factor on the basis of filter state abnormality judgment has stronger adaptability in practical engineering applications.

Experiments show that the algorithm in this paper can effectively suppress the divergence of filtering when the system noise is disturbed and, at the same time, has stronger robustness and self-adaptability compared with MFKF, which has certain reference significance for practical engineering applications.

## Figures and Tables

**Table 1:** IMU technical parameters.

IMU Parameter	Value
INS out frequency	100 Hz
Gyro bias	1°/h
Gyro angle random walk	5°/sqrt (h)
Accelerometer bias	50 μg

**Table 2:** INS/GNSS integrated navigation simulation positioning error.

Algorithm	Error Mean (m)			Error Standard Deviation (m)		
	North	East	Horizontal	North	East	Horizontal
KF	1.02	0.89	1.50	1.14	0.86	1.28
MFKF	0.66	0.64	1.02	0.55	0.50	0.59
AMFKF	0.59	0.54	0.89	0.46	0.45	0.51

**Table 3:** IMU parameters.

MEMS Parameter	Value
INS out frequency	100 Hz
Gyro bias	10°/h
Gyro angle random walk	5°/sqrt (h)
Accelerometer range	±5 g
Accelerometer bias	1 mg

**Table 4:** GNSS positioning error statistics.

Statistics	Position Error (m)		
	North	East	Horizontal
Mean	2.19	1.78	3.10
Max	10.68	5.39	10.82

**Table 5:** INS/GNSS integrated navigation positioning error.

Algorithm	Position Error (m)			Error Standard Deviation (m)		
	North	East	Horizontal	North	East	Horizontal
KF	2.83	2.26	3.99	2.64	2.26	3.05
MFKF	2.22	1.84	3.17	1.75	1.25	1.69
AMFKF	2.15	1.58	2.93	1.50	1.19	1.49

## Data Availability

Not applicable.
